# Supplementary material for: Laser-induced plasmonic colours on metals
Source: Nat Commun. 2017 Jul 18;8:16095. doi: 10.1038/ncomms16095 (PMC5520110; doi:10.1038/ncomms16095)
Supplement: Supplementary Information [file ncomms16095-s1.pdf]

Title of file for HTML: Supplementary Information
Description: Supplementary Figures, Supplementary Notes and Supplementary References

Title of file for HTML: Supplementary Movie 1
Description: Frontal view. Time evolution of a plane-wave interacting with the nanoparticles embedded by half their radii into the substrate.

Title of file for HTML: Supplementary Movie 2
Description: Lateral view. Time evolution of a plane-wave pulse interacting with the nanoparticles embedded by half their radii into the substrate.

Title of file for HTML: Peer Review File
Description:

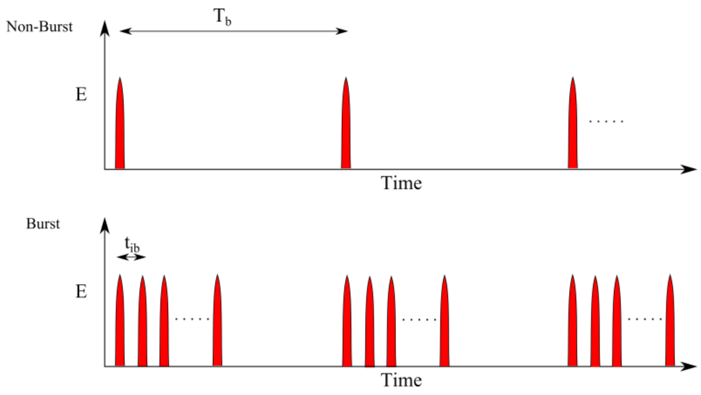

Supplementary Figure 1 | Schematic representation comparing the non-burst and burst operation of the machining laser. The repetition rate of the laser in normal operation (*i.e.*, non-burst) is defined by $f = 1/T_b$ where T_b is the burst period. In these experiments, f was fixed at 50 kHz. In burst mode a choice of 2 to 8 pulses could be chosen with a fixed time separation of t_{ib} = 12.8 ns. The energy of each pulse within the burst could be controlled via FlexBurst™.

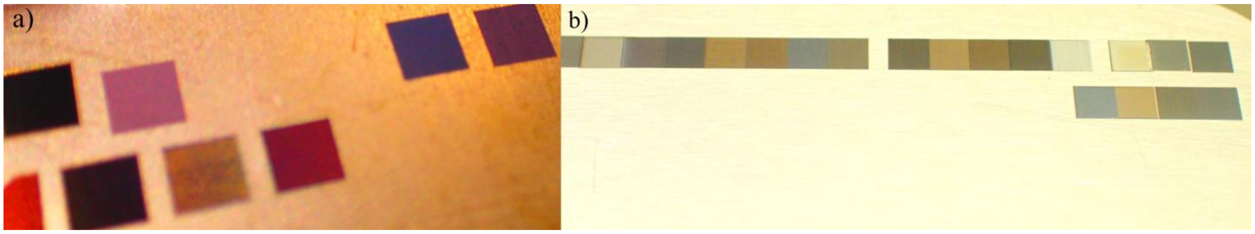

Supplementary Figure 2 | Photographs of colours obtained on (a) copper and (b) aluminum using the burst colouring method. The different colours were obtained by varying the spacing between successive laser lines.

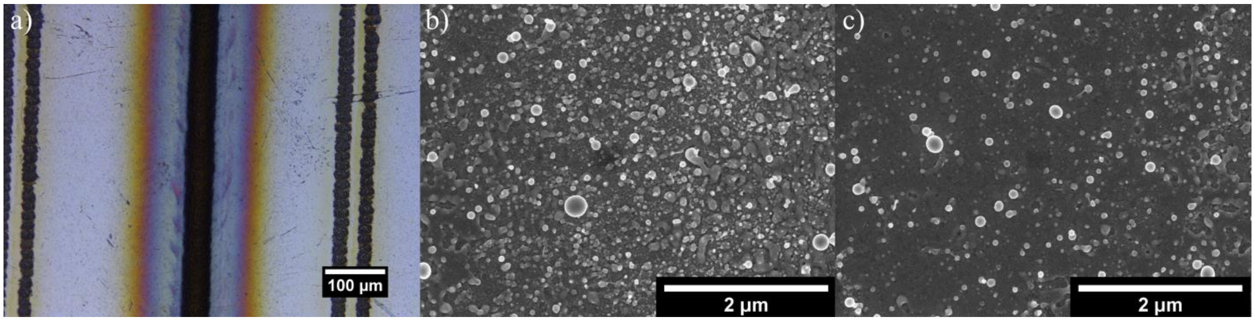

Supplementary Figure 3 | (a) Photograph of a laser ablated line on silver taken under 50x magnification. (b) SEM image of the nanoparticles deposited close to the ablated line shown in (a). (c) SEM image of the nanoparticles deposited further away from the ablated line shown in (a).

Supplementary Note 1 | Particle density distribution

The particle density resulting from the laser ablation of a single line was observed to differ along the direction normal to the line, Supplementary Figure 3 (b,c). The different particle densities can be inferred by the color gradient next to be ablated line, Supplementary Figure 3 (a). From this information the proper spacing between subsequent lines can determined in order to tune to the desired colour. In the case of Supplementary Figure 3 (a), a

very slow speed was used in order to compensate for the lack of subsequent lines which as a result produced a groove.

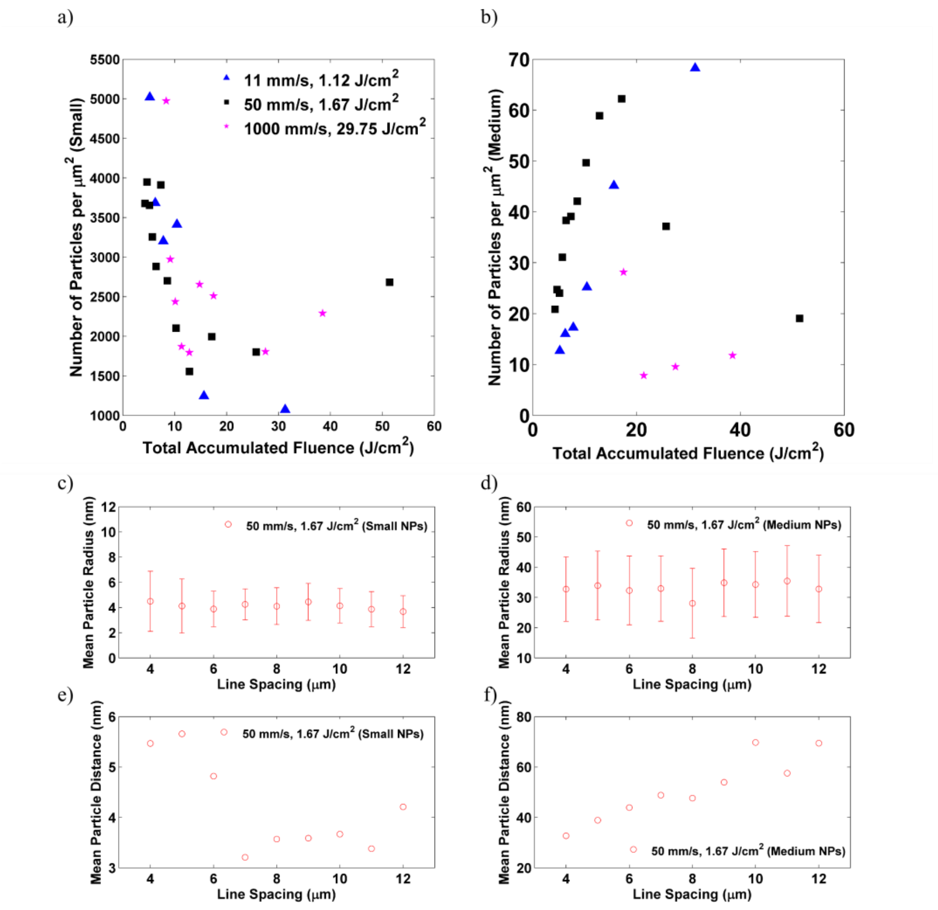

Supplementary Figure 4 | Number of particles per μm^2 versus total accumulated fluence for (a) small nanoparticles and (b) medium nanoparticles for a fluence φ = 1.12 J/cm^2 at laser marking speed v = 11 mm/s; φ = 1.67 J/cm^2 at v = 50 mm/s; and φ = 29.75 J/cm^2 at v = 1000 mm/s. Mean particle radius versus line spacing for (c) small NPs and (d) medium-large NPs for φ= 1.67 J/cm^2 at v = 50 mm/s. Mean inter-particle distance (wall-to-wall) versus line spacing for (e) small NPs and (f) medium-large NPs for φ = 1.67 J/cm^2 at v = 50 mm/s. The errors bars are standard deviation obtained from the statistical analysis of 3 SEM images within different areas of the same coloured squares.

Supplementary Note 2 | Particle Statistics

Surface analysis of coloured regions reveal that the number density of small particles, produced using different laser parameters, follows its own trend, Supplementary Figure 4 (a), similar to that of Fig. 4 (a), whereas the number density of the medium particles does not, Supplementary Figure 4 (b). This observation suggests that the small particles play a major role in the colours perceived. The mean radius of small and medium nanoparticles was found to remain approximately constant as a function of line spacing, as observed in Supplementary Figure 4 (c,d) (determined from the analysis of 3 SEM images per line spacing, *i.e.*, per colour). However, the mean inter-particle

(wall-to-wall) distance changes with line spacing, so the colours are believed to be caused by near-field interactions between nanoparticles in close proximity[1,2,3,4], particularly the associated surface plasmon resonance frequency[4].

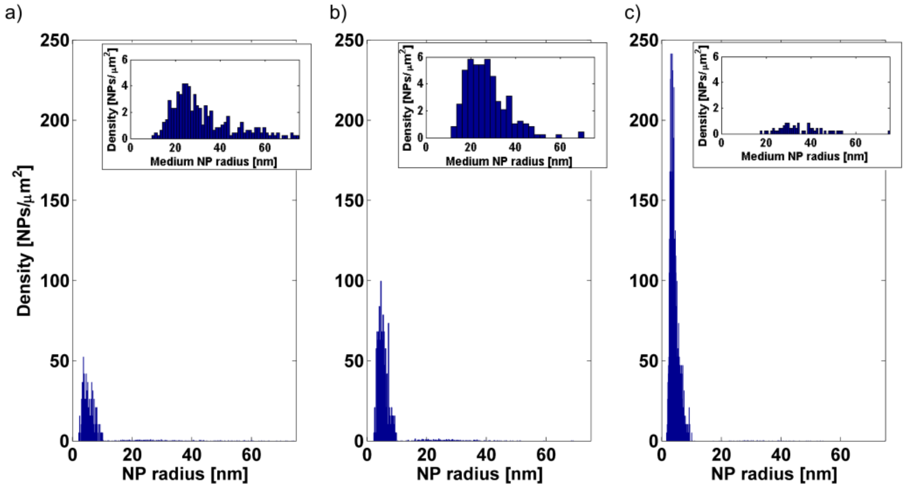

Supplementary Figure 5 | Histogram of the number of particles on the substrate relative to their radius over a surface area of 1 μm^2 for a fluence φ = 1.12 J/cm^2 and line spacing (a) L_s = 5 µm, (b) L_s = 10 µm, and (c) L_s = 30 µm, at a marking speed v = 11 mm/s. Two discernible bumps (bimodal distribution) are noted in the histograms, corresponding to the small and medium particles. The inserts are magnifications of the medium particle distributions.

Supplementary References

1. Rechberger, W. *et al.* Optical properties of two interacting gold nanoparticles. *Optics Communications* **220**, 137–141 (2003).
2. Romero, I., Aizpurua, J., Bryant, G. W. & Garcıa De Abajo, F. J. Plasmons in nearly touching metallic nanoparticles: singular response in the limit of touching dimers. *Optics Express* **14**, 9988–9999 (2006).
3. Jain, P. K. & El-Sayed, M. A. Plasmonic coupling in noble metal nanostructures. *Chemical Physics Letters* **487**, 153–164 (2010).
4. Liz-Marza, L. M. Tailoring Surface Plasmons trough the Morphology and Assembly of Metal Nanoparticles. *Langmuir* **22**, 32–41 (2006).
